# Sugammadex Versus Neostigmine: A Comparative Study of Efficacy and Safety in the Cath Lab

**DOI:** 10.7759/cureus.108066

**Published:** 2026-04-30

**Authors:** Pranav Prabhakaran, Sanjeeta Umbarkar, Girish Sabnis, Prerana Dixit

**Affiliations:** 1 Department of Cardiac Anaesthesia, Seth GS Medical College and King Edward Memorial Hospital, Mumbai, IND; 2 Department of Cardiology, Seth GS Medical College and King Edward Memorial Hospital, Mumbai, IND

**Keywords:** anesthesiology, cardiology, cath lab, neostigmine, sugammadex

## Abstract

Background

Effective and complete reversal of neuromuscular blockade is essential for safe recovery from general anesthesia. This becomes more significant in patients undergoing cardiac procedures as they have limited cardiopulmonary reserve and reduced tolerance for physiological instability. Neostigmine is the conventional agent of choice for the reversal of non-depolarizing neuromuscular blockade. However, its use is associated with variable efficacy and unwanted side effects such as bradycardia and bronchoconstriction. Sugammadex, a novel relaxant binding agent, reverses neuromuscular blockade by encapsulating free rocuronium molecules, thereby rapidly diminishing their functional presence at the neuromuscular junction. Compared with neostigmine, sugammadex has demonstrated accelerated and more predictable reversal, including from a deeper level of blockade.

Materials and methods

The current study is a prospective, randomized, triple-blinded interventional study done for a period of one year in a tertiary care hospital in India. Fifty adult patients scheduled for an elective cardiac catheterization procedure under general anesthesia were randomized into two equal groups (n=25 each) to receive either sugammadex or neostigmine for the reversal of neuromuscular blockade. The primary aim was to compare the efficacy and safety of sugammadex and neostigmine in reversing moderate neuromuscular blockade, defined as the reappearance of the second twitch (T2) on train-of-four (TOF) monitoring until the attainment of a TOF ratio of ≥0.9, in adult patients undergoing cardiac catheterization procedures under general anesthesia. Secondary objectives included evaluating adverse event profiles and time to discharge readiness from the post-anesthesia care unit (PACU) using the modified Aldrete score.

Results

The mean time from the reappearance of the second twitch (T2) on TOF monitoring until the attainment of a TOF ratio of ≥0.9 was significantly shorter in the sugammadex group as compared to the neostigmine group (t=14.42; p<0.001). Time to PACU discharge readiness was also significantly reduced with sugammadex (50.24±17.89 minutes) compared with neostigmine (65±15.45 minutes; t=3.15; p=0.002). Adverse effects occurred less frequently in the sugammadex group.

Conclusion

Sugammadex provides a swifter, more predictable, and safer reversal of neuromuscular blockade compared with neostigmine in patients undergoing cardiac catheterization procedures. Sugammadex facilitates earlier recovery and improved postoperative outcomes in the cath lab setting.

## Introduction

Muscle relaxation is an essential component of general anesthesia, facilitating endotracheal intubation and providing optimal surgical conditions. At the conclusion of the procedure, timely and effective reversal of neuromuscular blockade becomes crucial. Inadequate or delayed reversal may lead to residual weakness, which can lead to airway obstruction, hypoxia, and other postoperative pulmonary complications [1,2]. These consequences are particularly concerning in patients undergoing cardiac procedures as they have limited cardiopulmonary reserve and reduced tolerance for physiological instability.

Neostigmine is the common reversal agent for non-depolarizing neuromuscular blocking agents (NMBAs) due to its easy availability and low cost. It increases acetylcholine concentrations at the neuromuscular junction (NMJ) by inhibiting acetylcholinesterase. It is effective against all non-depolarizing NMBAs [3]. Its clinical utility is limited by several factors, including a ceiling effect, variable onset of action, and undesirable muscarinic side effects. Although glycopyrrolate is co-administered to mitigate these effects, the combination may still produce adverse reactions, including blurred vision and tachycardia [3,4]. Furthermore, neostigmine is less effective in reversing deep neuromuscular blockade, making it a suboptimal choice in scenarios requiring rapid and stable reversal.

Sugammadex is a novel reversal agent that aims to mitigate these issues. It acts by encapsulating rocuronium and vecuronium molecules, reducing their plasma concentration, and in turn causing dissociation from NMJ [5,6]. This mechanism allows rapid reversal despite the stage of block. Sugammadex has demonstrated favorable safety and efficacy profiles in patients with cardiac and respiratory comorbidities, showing reduced incidence of residual neuromuscular block and improved recovery outcomes [6]. Reported adverse effects of sugammadex include anaphylaxis, bradycardia, and coagulation abnormalities. In cardiac catheterization laboratories, transcatheter structural heart interventions, including those for atrial septal defects, ventricular septal defects, and ruptured sinus of Valsalva aneurysms, are commonly performed under general anesthesia with muscle relaxation especially in cases which require transesophageal echocardiography guidance [7]. Stable operating conditions, precise catheter manipulation, and rapid emergence from anesthesia are critically important in this setting. Faster and more predictable reversal with sugammadex also has the potential to reduce postoperative pulmonary complications, shorten intensive care unit (ICU) or post-anesthesia care unit (PACU) stay, and enhance procedural turnover rate, an important advantage in high-volume cardiac catheterization units. Although several studies have demonstrated the advantages of sugammadex over neostigmine in various surgical populations, prospective randomized data in this high-risk cohort remain limited [8]. Given these considerations, the present prospective randomized controlled trial was designed to compare these two drugs in a cath lab setup in terms of safety and efficacy. The primary aim was to find the time taken for the reversal of moderate neuromuscular blockade, defined as the reappearance of the second twitch (T2) to achieving a train-of-four (TOF) ratio of ≥0.9, in adult patients undergoing cardiac catheterization procedures. Secondary objectives included evaluating adverse event profiles and time to discharge readiness from the PACU using the modified Aldrete score.

## Materials and methods

This prospective, randomized, triple-blinded interventional study was conducted at Seth GS Medical College and King Edward Memorial Hospital in Mumbai, India, over a period of one year. The primary aim was to compare the efficacy and safety of sugammadex and neostigmine in reversing moderate neuromuscular blockade, defined as the reappearance of the second twitch on TOF monitoring until the attainment of a TOF ratio of ≥0.9, in adult patients undergoing transcatheter structural heart interventions under general anesthesia. Secondary objectives included evaluating adverse event profiles, hemodynamic stability, and time to discharge readiness from the PACU using the modified Aldrete score.

Patient selection

Patients aged 20-60 years, classified as American Society of Anesthesiologists (ASA) physical statuses II to IV, scheduled for elective cardiac catheterization procedures were screened for eligibility. Inclusion required written informed consent and willingness to participate.

Exclusion criteria

Criteria for exclusion were as follows: emergency procedures; inability or refusal to provide consent; known renal disease (estimated glomerular filtration rate (eGFR) less than 90 mL/min/1.73 m^2^ or serum creatinine >1.4), hepatic disease, or significant pulmonary disease; pre-existing neuromuscular disorders; and allergy to sugammadex, neostigmine, or glycopyrrolate.

Ethics committee approval was obtained from the Institutional Ethics Committee (IEC)-II Relating to Biomedical and Health Research (BHR) of Seth GS Medical College and King Edward Memorial Hospital (approval number: IEC(II)/OUT/162/2023) prior to study initiation, and clinical trial registration was done (Clinical Trials Registry-India (CTRI) number: CTRI/2023/06/053360).

Randomization and blinding

Fifty eligible patients were randomized into two groups (n=25 each) using a lottery method. This was a triple-blind study: the patient, the investigator administering the drug, and the observer recording outcomes were all blinded to group allocation. Another anesthesiologist, not involved in outcome assessment, prepared the study drugs according to patient weight and handed them to the investigator in identical syringes to maintain blinding.

The two groups were as follows: Group S (sugammadex), who received 2 mg per kg IV, and Group N (neostigmine), who received 0.05 mg per kg IV + glycopyrrolate 0.008 mg per kg IV.

Preoperative evaluation

A detailed history, general examination, and systemic evaluation were performed for all patients. Routine investigations included complete blood count, liver function tests, renal function tests, serum electrolytes, urine routine, and prothrombin time (PT)/international normalized ratio (INR).

Anesthesia technique

Patients were kept nil per os from midnight. Standard ASA monitors like electrocardiogram (ECG), noninvasive blood pressure, pulse oximetry, respiratory rate, and end-tidal CO2 temperature probe (nasopharynx) were attached.

Neuromuscular monitoring was done using the GE E-NMT module integrated with the GE Avance CS2 anesthesia workstation (GE HealthCare, Chicago, Illinois, United States). TOF stimulation was applied to the ulnar nerve using surface electrodes, and responses were recorded from the adductor pollicis muscle. The monitor was calibrated before giving a muscle relaxant to the patient to determine the supramaximal stimulus. Nasopharyngeal temperature was maintained above 35°C throughout the case to ensure the reliability of TOF measurements as hypothermia can prolong neuromuscular blockade and affect TOF readings.

Induction and maintenance

Premedication included glycopyrrolate 0.2 mg IV and midazolam 1 mg IV. Analgesia was provided with fentanyl 2 micrograms/kg IV, and ondansetron 0.01 mg/kg IV was administered for antiemetic prophylaxis. Anesthesia was induced with etomidate 0.2 mg/kg IV, and neuromuscular blockade was achieved with rocuronium 1 mg/kg to facilitate endotracheal intubation. Anesthesia was maintained with 50% oxygen, 50% air, and sevoflurane via closed-circuit intermittent positive-pressure ventilation. Additional rocuronium doses (0.1 mg/kg) were administered as required.

Reversal protocol

After the completion of the procedure, defined as the completion of interventional steps and the removal of all catheters, quantitative TOF neuromuscular monitoring was continued. Upon the appearance of the second twitch of the TOF monitor, patients received the allocated reversal agent. The time taken to achieve a TOF ratio of ≥0.9 was recorded.

Patients were monitored from induction until achieving discharge readiness in the PACU.

Rescue reversal

If sugammadex failed to achieve a TOF ratio of ≥0.9 within a reasonable timeframe, reversal was completed with neostigmine/glycopyrrolate per standard practice.

Outcome measures

The primary outcome was the time from the administration of the reversal agent to the achievement of a TOF ratio of ≥0.9. Secondary outcomes were the incidence of adverse events, hemodynamic stability, and time to PACU discharge readiness.

Adverse events were predefined as any complications occurring from the time of administration of the study drug till discharge from PACU including bradycardia (heart rate <50 beats per minute), hypotension (mean arterial pressure <60 mmHg), hypoxia (SpO2 <92%), airway obstruction, residual neuromuscular blockade, postoperative nausea and vomiting, and hypersensitivity reactions. Hemodynamic stability was assessed by measuring mean arterial blood pressure at regular intervals with intervention required if it drops below 60 mmHg. Time to discharge readiness was defined as the time from reversal to achieving a modified Aldrete score of more than or equal to 9, assessed at five-minute intervals. 

Sample size calculation

Sample size was calculated using OpenEpi 2.3.1 (Dean AG, Sullivan KM, Soe MM. OpenEpi: Open Source Epidemiologic Statistics for Public Health, Version. www.OpenEpi.com, updated 2013/04/06), an open-source statistical online tool. According to a study by Mahajan and Singh [9], the mean time of PACU stay in minutes in the sugammadex group was 38.0±9.0, while it was 52.0±12.0 in the neostigmine group, with a mean difference of 14.0, a confidence interval of 99%, and a power of 95%. The sample size was calculated to be 21 in each group, which was rounded off to 25 in each group.

Statistical analysis

Data were analyzed using Epi Info Version 7.2 (Centers for Disease Control and Prevention, Atlanta, Georgia, United States), a free online statistical software package.

Quantitative variables were expressed as mean±SD and compared using Student's t-test. Qualitative variables were expressed as percentages and analyzed using the chi-squared test. A p-value of <0.05 was considered statistically significant.

**Figure 1:**
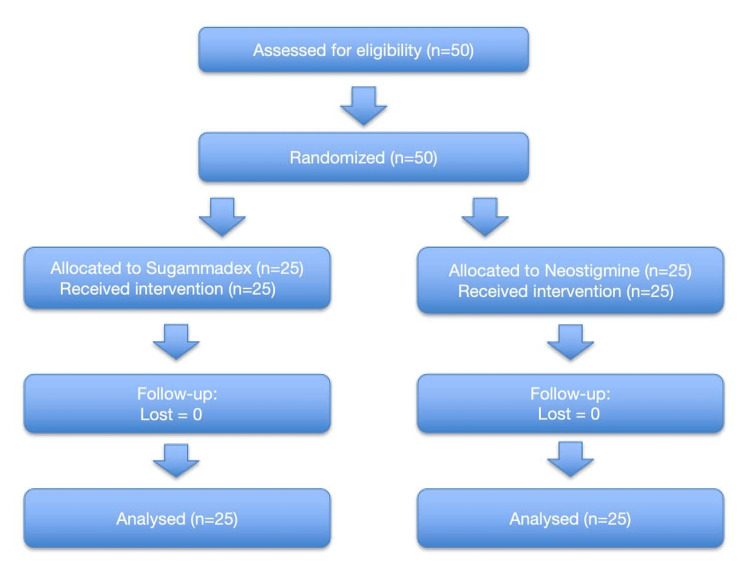
CONSORT flow diagram CONSORT: Consolidated Standards of Reporting Trials

This study was conducted and reported in accordance with the Consolidated Standards of Reporting Trials (CONSORT) guidelines (Figure 1).

## Results

Table 1 summarizes the demographic and clinical characteristics of participants in both groups. The mean age was 39.45±8.25 years in the neostigmine group and 36.24±4.79 years in the sugammadex group (p=0.098). The mean weight was also comparable between groups (54.51±11.42 kg vs. 50.22±8.94 kg; p=0.144). Gender distribution did not differ significantly (male: 48% vs. 36%; p=0.195). Most participants belonged to ASA class III in both groups (92% vs. 84%; p=0.192). The distribution of surgical procedures was similar, with atrial septal defect device closure performed in 92% of the neostigmine group and 84% of the sugammadex group (p=0.192). Overall, the groups were well-matched, with no statistically significant differences in baseline characteristics.

**Table 1:** Baseline characteristics and ASA grading An unpaired Student's t-test was used to compare the means and standard deviation. A p-value of <0.05 was considered statistically significant. The chi-squared test was used to find the association. ASA: American Society of Anesthesiologists; ASD: atrial septal defect; RSOV: ruptured sinus of Valsalva

Variable	Neostigmine	Sugammadex	T-value	P-value
	Mean±SD	Mean±SD		
Age (years)	39.45±8.25	36.24±4.79	1.682	0.098
Weight (kg)	54.51±11.42	50.22±8.94	1.484	0.144
			Chi square	P-value
Gender				
Male	12 (48%)	9 (36%)	0.738	0.195
Female	13 (52%)	16 (64%)		
Total	25	25		
ASA grading				
II	2 (8%)	4 (16%)	0.75	0.192
III	23 (92%)	21 (84%)		
Total	25	25		
Type of surgery				
ASD device closure	23 (92%)	21 (84%)	0.75	0.192
RSOV device closure	2 (8%)	4 (16%)		
Total	25	25		

The primary outcome, defined as the time from the reappearance of T2 to achieving a TOF ratio of ≥0.9, was significantly shorter in the sugammadex group compared to the neostigmine group as shown in Table 2. The mean time to recovery was 70.56±24.45 seconds with sugammadex versus 270.25±54.8 seconds with neostigmine (p<0.001), indicating an approximately fourfold faster reversal. Similarly, for the secondary outcome, time to PACU discharge readiness as assessed by the modified Aldrete score was significantly reduced in the sugammadex group. The mean time to achieve discharge readiness was 50.24±17.89 minutes with sugammadex compared to 65.12±15.45 minutes with neostigmine (p=0.002), demonstrating earlier postoperative recovery.

**Table 2:** Time from T2 to TOF ≥0.9 in seconds and time taken for discharge readiness from PACU in minutes An unpaired Student's t-test was used to compare the means and standard deviation. A p-value of <0.05 was considered statistically significant. T2: second twitch; TOF: train-of-four; PACU: post-anesthesia care unit

Variable	Neostigmine	Sugammadex	T-value	P-value
	Mean±SD	Mean±SD		
Time from T2 to TOF ≥0.9 (seconds)	270.25±54.8	70.56±24.45	14.42	<0.001
Time taken for discharge readiness from PACU (minutes)	65.12±15.45	50.24±17.89	3.15	0.002

As shown in Table 3, 28% of patients in the neostigmine group experienced adverse effects compared with 8% in the sugammadex group, indicating a more favorable safety profile for sugammadex.

**Table 3:** Side effects in both groups

Variable	Neostigmine	Sugammadex
Postoperative nausea vomiting	4 (16%)	2 (8%)
Urinary retention	2 (8%)	0
Shortness of breath	1 (4%)	0

The mean arterial pressure trends at various time intervals are shown in Table 4. Although the neostigmine group demonstrated slightly higher mean arterial pressure values at most intervals, none of the differences were statistically significant (p>0.05 at all time points). Both groups maintained stable hemodynamics throughout the study.

**Table 4:** Comparison of mean blood pressure in both groups An unpaired Student's t-test was used to compare the means and standard deviation. A p-value of <0.05 was considered statistically significant.

Time in minutes	Neostigmine	Sugammadex	P-value	Significance
	Mean±SD	Mean±SD		
2 minutes	63.87±9.74	61.93±8.68	0.569	Not significant
4 minutes	62.60±9.73	60.33±10.78	0.549	Not significant
6 minutes	62.47±9.16	62.4±10.75	0.984	Not significant
8 minutes	69.20±9.23	68.8±9.23	0.784	Not significant
10 minutes	67.40±9.93	64.6±8.27	0.854	Not significant
15 minutes	67.87±9.23	63.73±8.47	0.559	Not significant
20 minutes	67.13±9.18	62.46±7.62	0.140	Not significant
30 minutes	66.67±8.18	64.13±5.70	0.333	Not significant
60 minutes	66.18±7.57	64.28±5.41	0.384	Not significant
90 minutes	65.43±5.86	63.8±5.30	0.456	Not significant

## Discussion

In this prospective randomized controlled trial, we compared the efficacy and safety of sugammadex and neostigmine for the reversal of moderate neuromuscular blockade in patients undergoing cardiac catheterization procedures. The primary outcome, time to achieve a TOF ratio of ≥0.9 following the reappearance of T2, was significantly shorter in the sugammadex group compared to the neostigmine group (70.56±24.45 seconds vs. 270.25±54.8 seconds).

Several other studies also report superior reversal times with sugammadex across varying depths of neuromuscular blockade [10-12]. Sacan et al. [13] similarly demonstrated quicker times to achieve TOF ratios of 0.7, 0.8, and 0.9 with sugammadex relative to neostigmine. Additionally, sugammadex has been shown to reliably reverse even deep neuromuscular blockade (1-2 PTC responses), with up to 98% of patients achieving a TOF ratio of ≥0.9 within five minutes, compared to 11% with neostigmine [14,15]. Grintescu et al. also demonstrated markedly faster recovery with sugammadex and reported that operating room occupancy time was significantly reduced in the sugammadex group [16]. Our findings support these observations. Faster neuromuscular recovery leads to an earlier achievement of PACU discharge readiness in our study, with patients receiving sugammadex demonstrating a mean PACU time approximately 15 minutes shorter than those receiving neostigmine. This is consistent with Carron et al.'s meta-analysis, which showed that sugammadex significantly reduces OR-to-PACU transfer time and speeds PACU discharge readiness [17].

Similar findings have been echoed in multiple earlier studies [18,19]. Several mechanisms may explain these results. Sugammadex's mechanism of action, which involves encapsulation, directly yields a predictable and rapid reversal profile. In contrast, neostigmine increases acetylcholine availability indirectly, relies on competitive displacement at the NMJ, and exhibits a ceiling effect. The afferentation theory further suggests that rapid restoration of neuromuscular function increases sensory input to the reticular activating system, thereby facilitating smoother and faster emergence from anesthesia [18,19].

In our study, the mean arterial pressure and heart rate remained comparable between groups without statistically significant differences. However, other authors have reported that neostigmine may cause transient increases in heart rate and blood pressure, whereas sugammadex maintains a more stable profile [8]. The absence of significant hemodynamic disturbances reinforces the suitability of sugammadex for cardiac patients, who often have limited tolerance for physiologic variability.

Adverse effects were more frequent in the neostigmine group in our study. Khuenl-Brady et al. [20] noted that the incidence of postoperative muscle weakness was similar in both sugammadex and neostigmine groups. Adverse effects like postoperative nausea and vomiting, urinary retention, and transient dyspnea occurred more frequently in the neostigmine group. These findings are consistent with the known muscarinic and gastrointestinal side effects of neostigmine and glycopyrrolate coadministration [21,22]. Anticholinergics, by inhibiting detrusor muscle contraction, likely contributed to the urinary retention observed in our patients [23]. Conversely, sugammadex does not act on acetylcholinesterase or muscarinic receptors and is therefore associated with fewer cholinergic adverse effects. Only postoperative nausea and vomiting occurred in the sugammadex group (8%), consistent with earlier safety profiles [24,25]. Although rare, reports of bradycardia, partial thromboplastin time (PTT) prolongation, and hypersensitivity with sugammadex have been documented [26,27]; none occurred in our sample.

The findings of this study are particularly relevant in the context of cardiac catheterization, where rapid emergence, minimal hemodynamic perturbation, and predictable reversal are crucial. Delayed recovery or incomplete neuromuscular reversal in this population can increase the risk of hypoxia, aspiration, arrhythmias, and prolonged procedural time. By enabling fast, reliable neuromuscular recovery, sugammadex may mitigate these risks and support smoother postoperative transitions.

The major factor impeding the popularity of sugammadex in Indian setups is the high cost of acquisition of the drug. But our study hints that this might be offset by the improved clinical profile of sugammadex. Although a formal cost-effectiveness analysis was beyond the scope of this study, sugammadex has the potential to lower overall perioperative costs by reducing PACU time, shortening anesthesia emergence, and decreasing postoperative pulmonary complications. In high-turnover environments such as cardiac catheterization laboratories, these improvements may significantly enhance workflow and patient throughput.

Limitations

Limitations include the small sample size, the single-center design, the lack of a long-term outcome assessment, the evaluation limited to moderate neuromuscular blockade only, and the exclusion of patients with severe renal dysfunction which limits generalizability. The cardiologist's variation in skills and experience may have influenced the time between procedure completion time and extubation, as well as the time between administering neuromuscular blockade reversal and extubation. This may have had an effect on the amount of time that passed between administering neuromuscular blockade and being discharged from the cath lab. Future studies with standardized procedural protocols could help minimize these variations and provide a more precise comparison.

## Conclusions

Our study confirms that sugammadex provides a faster, more predictable, and safer recovery profile compared to neostigmine for neuromuscular blockade reversal in cardiac procedures performed in the cath lab. While previous studies have largely focused on operating room settings, this study aimed to evaluate neuromuscular blockade reversal in the cardiac catheterization lab, a setting characterized by hemodynamically fragile patients, shorter procedural duration, and the need for rapid recovery. By specifically assessing reversal at a clinically relevant endpoint, this study provides practical evidence supporting the preferential use of sugammadex in this setting. 
